# EFFECTS OF PHYSIO-FEEDBACK EXERCISE PROGRAM (PEER) ON PHYSICAL ACTIVITY LEVELS IN OLDER ADULTS

**DOI:** 10.1093/geroni/igae098.3480

**Published:** 2024-12-31

**Authors:** Jethro Raphael Suarez, Joon-Hyuk Park, Rui Xie, Kworweinski Lafontant, Ladda Thiamwong

**Affiliations:** University of Central Florida; University of Central Florida, Orlando, Florida, United States; University of Central Florida, Orlando, Florida, United States; University of Central Florida, Orlando, Florida, United States; University of Central Florida, Orlando, Florida, United States

## Abstract

Adults aged 60 years and older are the most sedentary group in the United States. Low levels of physical activity (PA) and high levels of sedentary behavior (SB) increase the risk for developing diseases. Therefore, it is crucial to investigate intervention strategies that help increase PA and decrease SB levels in older adults. This study was an 8-week, Physio-fEedback Exercise pRogram (PEER), aimed at improving PA levels and balance while reducing the fall risk of community-dwelling older adults by providing visual physio-feedback on balance and body composition, cognitive reframing to lower the fear of falling, and a peer-led group exercise. We conducted a preliminary analysis of 55 community-dwelling older adults aged 61 to 89 years (49 women, 6 men; mean age = 73.75 ± 7.28 years) in low-income settings to determine if the intervention influenced PA levels. Total PA levels were measured across 7 consecutive days using the ActiGraph GT9X Link wireless activity monitor at baseline and post-intervention for a control (n = 31) and intervention group (n = 24), and categorized as sedentary behavior (SB), light PA (LPA), and moderate-to-vigorous PA (MVPA). PA was compared between groups using Student’s t-test and Mann-Whitney U test, but significant differences were not found. However, the PEER group showed a decrease in total SB mean (208.64 min), increase in total LPA mean (89.99 min), and increase in total MVPA median (8.75 min) between baseline and post-intervention. The results show that PEER has the potential to impact PA levels, but more research is warranted.

